# Effect of Chitosan/Gum Arabic Blends Enriched by Sodium Nitroprusside or Methyl Salicylate on the Storability and Antioxidant Activity of Tomato Fruit

**DOI:** 10.3390/polym16111518

**Published:** 2024-05-28

**Authors:** Mohamed A. Taher, Dawood H. Dawood, Mohammed A. E. Selim, Basma H. Amin, Elsherbiny A. Elsherbiny

**Affiliations:** 1 Agricultural Chemistry Department, Faculty of Agriculture, Mansoura University, Mansoura 35516, Egypt; dhosni@mans.edu.eg; 2 Agricultural Microbiology Department, Faculty of Agriculture, Mansoura University, Mansoura 35516, Egypt; maalawady@mans.edu.eg; 3 Regional Center for Mycology and Biotechnology (RCMB), Al-Azhar University, Cairo 11651, Egypt; dr_bas76_azh@yahoo.com; 4 Plant Pathology Department, Faculty of Agriculture, Mansoura University, Mansoura 35516, Egypt

**Keywords:** methyl salicylic acid, sodium nitroprusside, fruit pigments, lipid peroxidation, antifungal activity, electron microscope

## Abstract

The impact of methyl salicylate (MeSA) or sodium nitroprusside (SNP) in chitosan (CS)/Gum Arabic (GA) mixture on physio-chemical characteristics and antioxidant status during the postharvest ripening of green tomato fruits was studied. CS/GA-MeSA at a 1 mM formulation was the best treatment to retard firmness and titratable acidity (TA) losses. Moreover, this formulation retarded pigmentation progress where it had the lowest significant values of total carotenes (TCs) and lycopene (LYP) contents until the 15th day of the storage period, as well as efficiently faced the rise in malondialdehyde (MDA) levels. Moreover, peroxidase (POD), polyphenol oxidase (PPO), catalase (CAT), and phenylalanine ammonia-lyase (PAL) activities of tomatoes treated with CS/GA-SNP at 2 mM were significantly better than that of control in the primary stages of storage. CS/GA-SNP at a 2 mM formulation showed an extremely high significant content of total polyphenol (TP) in the early stage of storage, while CS/GA and CS/GA-MeSA at 1 and 2 mM accumulated higher significant TP contents than uncoated fruits at the late stage of storage. All formulations were characterized by FTIR spectroscopy. Furthermore, the polymer formulations exhibited strong antifungal activity against *Alternaria alternata* and *Botrytis cinerea* as major pathogens of postharvest tomatoes. Transmission electron microscope (TEM) observations for the mycelia of both fungi treated by CS/GA-MeSA at 2 mM revealed serious ultrastructural damage, including distortion of the cell wall and cell membrane and degradation of cytoplasmic organelles.

## 1. Introduction

Egypt faces momentous challenges associated with climate change, urbanization, and rapid population growth along the Nile River, as well as water and food security issues [1]. In this respect, the local government began in 2016 to cultivate desert land in Marsa Matrouh, Sinai, and 10th of Ramadan City with complexes of water-preserving greenhouses via the national greenhouse project to face an increased need for vegetables in the Egyptian market [2]. Moreover, several factories for packaging agricultural products have been constructed in several locations around greenhouse areas. Greenhouse cultivation shields various vegetable crops from extreme climate conditions, especially high humidity and temperatures, resulting in a higher yield of fruits with rich water and nutrient content, and offering the benefit of off-season growth [2,3].

In the case of tomato, its fruit is globally cultivated throughout the year for salad-making, cookery purposes, and in producing ketchup and dried powders [4]. It holds attractive health elements such as ascorbic acid, phenolics, lycopene, and β-carotene [4]. Unfortunately, tomato quickly ripens and decay, particularly in summer with high temperatures. Moreover, the desert climate conditions in summer very greatly increase the susceptibility of tomato fruit to pathogenic fungi, particularly in the shelf-life period, and, therefore, accumulates a high content of reactive oxygen species (ROS) and causes the oxidation of phenolics, quickening their degeneration [5]. The huge ROS level affects membrane lipid peroxidation (LPOX), which causes constant injury to the cell membrane [6]. Based on previous evidence, the open field cultivation of tomatoes in Egyptian desert land is not valuable due to the poor yield with low quality. Moreover, the greenhouse cultivation of tomatoes in desert lands imposed its conversion into processed products due to the great distance between the cultivation and consumption area in the delta and Nile Valley, theoretically causing an extreme deterioration in fruit quality standards like weight loss, fungal infections, and the loss of flavors and aromas. The Egyptian kitchen mostly favors fresh tomatoes over processed products based on the criteria. Therefore, the good supply of fresh tomatoes from new lands into the old local market critically depends on harvesting in the green-stage period and preserving them in controlled conditions to decline shelf-life deterioration [5,7]. In this regard, the use of coatings of biodegradable polymers or their blends has elevated the selective permeability of the water/gas interface causing respiratory control and delaying damaging signs like anaerobic fermentation and the development of aging, in addition to the inhibition of microbial growth accountable for fruit spoilage [8].

Chitosan (CS) is a linear polycationic natural polysaccharide derived from chitin partial deacetylation. It is the second most abundant organic material after cellulose in nature [9]. Structurally, it contains β-(1→4)-N-acetyl-d-glucosamine and β-(1→4)-d-glucosamine moieties [10]. It is a promising material due to its degradability, non-toxicity, biocompatibility, and antimicrobial potential [9]. CS is widely recognized for its use in fabricating biodegradable films, gels, hydrogels, and CS-based nanocomposite films [11]. Consequently, CS is regarded as a promising substance for creating membranes suitable for various uses, including fruit preservation [10,12]. Unfortunately, the low mechanical strength property of CS limits the efficacy of its coating films [8]. Hence, CS blends with a high mechanical strength property is a perfect tactic for improving the mechanical traits of CS.

Gum Arabic (GA) is another natural polyanionic polysaccharide obtained from the bark of *Acacia senegal*. Structurally, arabinogalactan is the key constituent of GA (80–90%) [13]. It has been permitted as a secure food additive for emulsification, stabilization, and micro-encapsulation [14]. GA is extensively used as an edible coating in the postharvest technology of fruits [15,16,17]. Moreover, a blend consisting of GA/CS was also itemized to enhance fruit quality in bananas [18]. Recently, a GA and polyvinylpyrrolidone (PVP) blend enriched with salicylic acid has been described to improve the postharvest period of peach fruit [14]. The polyanionic GA can interact with polycationic polymers such as CS [19]. CS/GA films are expected to exhibit improved functional properties. Moreover, studies have shown that incorporating plant essential oils into CS/GA films enhances their thermal, physical, and antimicrobial characteristics [19]. Moreover, CS/GA films enriched with thyme oil were efficiently used to encapsulate blood orange anthocyanins, improving their multi-functionality [20].

Salicylic acid (SA) and its derivative methyl salicylate (MeSA) are currently designated as hormonal components keeping variable physiological properties on plant growth, particularly during biotic stresses due to their ability to induce systemic acquired resistance [21,22]. Moreover, the postharvest application of MeSA reduced chilling injury and decay and improved fruit quality attributes, such as texture, appearance, and nutritional content within the fruit supply chain [23]. MeSA is a volatile compound produced endogenously by plants in response to herbivores’ attacks [24]. Previous reports have evidenced the repellent impact of MeSA on agricultural pests by altering their performance via reducing behavior, oviposition, and colonization [25]. The application of MeSA on plant tissues has been evaluated in many forms, such as trap cards, lures, and direct applications via fumigation and foliar spray [24,26,27]. Recently, Lee et al. [28] fabricated, for the first time, a bioactive film suitable for food packaging consisting of β-cyclodextrin and MeSA with the controlled release of that volatile compound. To our knowledge, up to now, no reports have focused on MeSA efficacy when incorporated in a biodegradable polymer film on postharvest attributes of different fruits or vegetables.

Nitric oxide (NO) is a highly reactive free radical gas produced for signaling proposes in plants that trigger defense responses to different biotic and abiotic stresses [29]. NO levels elevated sharply due to plant–pathogen interaction initiating resistance against plant diseases [30]. The endogenous concentrations of NO and ethylene during the growth and maturation of the fruits have opposite and stoichiometric relationships. The NO concentration declines with repining progress and senescence in different horticultural crops [31], thus offering the chance to increase its level with exogenous treatment to exert the contrary effect. Therefore, the impacts of exogenous sodium nitroprusside (SNP) treatment as an NO donor in the disease resistance and senescence diminishing of several fruits have gained much attention in recent years. In this regard, earlier reports exhibited that NO efficiently improved tomato fruit resistance to gray mold rot [32] and some other fruits’ resistance to anthracnose [30,33,34]. Numerous studies indicate that NO may be a viable and economical substitute for synthetic fungicides [35]. Furthermore, NO fumigation is reported to postpone the ripening of Japanese plums and reduce chilling injury during cold storage [31]. Generally, the application of NO gas has been typically performed as a fumigant or released from an aqueous solution containing one of its donor compounds such as S-nitrosothiols, diazeniumdiolates, and SNP also applied as a dipping treatment [36]. The synergistic effect of NO treatment in combination with cold storage or modified atmosphere conditions in extending the storage life of different fruits has been stated [31,37,38]. The improvement of the postharvest period and antioxidant status of sweet cherry fruit using NO-liberating CS nanoparticles has been reported by Ma et al. [39]. They used nitrosated glutathione as an NO donor in the CS nanoparticle solution. To the best of our knowledge, the impacts of polymeric-coating materials enriched by SNP as an NO donor on quality attributes of postharvest fruits have not been estimated yet.

The present study aims to evaluate the effect of different composites based on the CS/GA polymer blend enriched by SNP or MeSA elicitors on ripening development, antioxidant status, and the induction of resistance in green mature tomato fruits cultivated in greenhouses and freshly harvested. It also aims to recommend the optimal treatment for freshly harvested tomatoes in new lands to guarantee a consistent supply of fresh tomatoes in the traditional markets of the delta and Nile Valley.

## 2. Materials and Methods

### 2.1. Chemicals

The subsequent chemical compounds were purchased from Sigma-Aldrich (St. Louis, MO, USA): catechol, 2,6-dichlorophenol indophenol (DCPIP), ethylenediaminetetraacetic acid (EDTA), Folin–Ciocalteu reagent, gallic acid, guaiacol, methyl salicylic acid (MeSA), phenylalanine, polyvinylpolypyrrolidone (PVPP), sodium nitroprusside (SNP), and thiobarbituric acid. Gum Arabic (GA, mol. wt. of approx. 250,000, purity > 98%), trichloroacetic acid, hydrochloric acid (HCl), boric acid, acetic acid, potassium hydroxide, butylated hydroxytoluene (BHT), ascorbic acid, sodium carbonate, sodium nitrite, sodium bicarbonate, hydrogen peroxide, phosphate salts for buffers, and organic solvents were obtained from El-Gomhoria Company for pharmaceutical chemicals, Egypt. Chitosan with MW of 71.3 kDa and 94% degree of deacetylation was obtained from Merck (Darmstadt, Germany).

### 2.2. Tomatoes

Agyad 7 genotype tomato seeds were cultivated in local greenhouse in Mansoura University, Mansoura city, Egypt. Fruits at the green stage of maturity were harvested. The uninfected or mechanically injured fruits with uniform shapes and sizes were selected for the trial.

### 2.3. Polymers Solutions

CS solution (1%) was used by dissolving 10 g CS in 1% acetic acid (1 L) with magnetic stirring for 2 h. Thirty grams of GA were dissolved in 1 L of distilled water in a hot water bath for 1 h for whole solvation to produce a 3% GA solution. CS/GA polymer blends (500 mL for each one) were obtained by mixing 250 mL of each of the earlier solutions for 2 h using a magnetic mixer at 60 °C. Sodium nitroprusside (SNP) and methyl salicylic acid (MeSA) at different concentrations were initially dissolved in 2 mL of each distilled water or Tween 80, respectively, followed by loading in 500 mL of CS/GA blend under magnetic stirring for 2 h at room temperature. Finally, the production of NO by SNP (1 and 2 mmol·L^−1^) in CS/GA blend solutions was assessed by determining NO end product utilizing Griess reagent (0.1% N-(1-naphthyl)-ethylenediamine and 1% sulfanilamide in H_3_PO_4_) [39]. Initially, the CS/GA-SNP 1 mM and CS/GA-SNP 2 mM solutions were immediately decomposed for 0, 30, 60, 120, and 180 min under the activation of visible light, and 2 mL of each blend aqueous solution was added into 2 mL of Griess reagent in a test tube maintained then at 25 °C for 20 min. The absorbance was recorded at 540 nm, and the NO concentrations were calculated by using the standard curve equation of NaNO_2_ and expressed as mmol L^−1^. Aqueous solutions of SNP (1 and 2 mM) were used as control.

### 2.4. Characterization of Tested Films

#### 2.4.1. Preparation of Coating Films

Fifty mL of each composite solution was poured into a Petri dish to obtain a thickness of about 60 µm and was left to dry at room temperature. Then, the resultant films were peeled from the dish and finally powdered.

#### 2.4.2. Attenuated Total-Reflectance Fourier-Transform Infrared Analysis (ATR-FTIR)

FTIR spectra of pure CS, GA, SNP, MeSA, and composite powders of CS/GA, CS/GA-SNP 2 mM, and CS/GA-MeSA 2 mM were performed over a wavenumber range of 4000 to 500 cm^−1^ using a Bruker Alpha spectrometer, supplemented with a total-reflection diamond-crystal accessory (Bruker Corporation, Rheinstetten, Germany) maintaining a resolution of 4 cm^−1^ with 32 scans.

### 2.5. Antifungal Activity

The antifungal activity of CS/GA, CS/GA-SNP 1 mM, CS/GA-SNP 2 mM, CS/GA-MeSA 1 mM, and CS/GA-MeSA 2 mM formulations against *Alternaria alternata* AUMC10301 and *Botrytis cinerea* AUMC 6095 (AUMC, Assiut University Mycological Centre, Assiut, Egypt) was evaluated in vitro [7]. The concentration of CS and GA in the medium was 1 and 2 g/100 mL, respectively.

### 2.6. Transmission Electron Microscope (TEM)

Mycelial samples were taken from PDA cultures embedded with CS/GA-MeSA 2 mM for both fungi, *A. alternata* and *B. cinerea*, and the cultures without CS/GA-MeSA were used as a control. The samples were fixed in glutaraldehyde (3%), rinsed in phosphate buffer, and post-fixed in potassium permanganate solution at room temperature for 5 min. The samples were dehydrated in an ethanol series (from 10% to 90%) for 15 min in each alcohol dilution and, finally, with ethanol 100% for 30 min. TEM samples were infiltrated with epoxy resin and acetone using a graded series and, finally, embedded in pure epoxy resin. Ultrathin sections were collected on copper grids and then stained in uranyl acetate followed by lead citrate. The hyphae of both pathogens were observed using a transmission electron microscope (JEM 1010, JEOL, Tokyo, Japan) at 80 kV.

### 2.7. Treatment of Tomato Fruits

Tomato fruits underwent treatment with NaClO (0.06% w/v) for 5 min, were then dipped in sterile water, and were subsequently air-dried at room temperature within a laminar flow cabinet. The fruits were separated into 6 groups (50 for each and 3 replicates); the fruits of the first group were considered as control, and thus dipped in distilled water for 5 min. The second set was immersed in the CS/GA blend at the same time. The fruits of the groups (3–6) were separately dipped into CS/GA-SNP 1 mM, CS/GA-SNP 2 mM, CS/GA-MeSA 1 mM, and CS/GA-MeSA 2 mM formulations, respectively, for 5 min. Subsequently, the fruits were air-dried at room temperature and stored in transparent folding PET boxes containing some holes at 90% relative humidity for 20 days at room temperature. The examined samples were taken at zero time and after 5, 10, 15, and 20 days.

### 2.8. Physical and Chemical Properties

#### 2.8.1. Fruit Firmness and Titratable Acidity

Fruit firmness as N units was noted using an Effegi-penetrometer accompanied by a plunger 8 mm-diameter penetrator [40]. Titratable Acidity (TA) as mg citric acid g^−1^ was assessed according to Taher et al. [4].

#### 2.8.2. Analysis of Fruit Pigments

Development of fruit color during repining can be monitored by extraction and determination of total caroteins (TCs) according to Carvalho et al. [41] and lycopene content (LYC) affording the procedure of [7]. In the TC test, two grams of tomato homogenate were extracted with 10 mL acetone, and the solid material was repeatedly treated with the same solvent until the tissue became colorless indicating full extraction of TCs. The acetone was combined in a separatory funnel followed by the addition of petroleum ether (PE) at 40–60 °C in a volume of 25 mL. Then, distilled water was added to the funnel mixture with a sensibly shaking. After equilibrium, the PE layer was separated, and the aqueous acetone layer was then retreated again with PE. The resultant PE layers were combined and treated with anhydrous sodium sulfate pellets to eliminate water traces. The absorbance of the PE phase was recorded at 450 nm; its value was then used to calculate TCs contents as µg g^−1^. On the other hand, a 1 g tomato homogenate sample was placed in a brown glass vial containing 0.04% (w/v) BHT/acetone, 95% ethanol, and hexane in a ratio of (1:2:4, v/v/v) for LYC extraction. The previous mixtures were then shaken for 20 min at 180 rpm. Then, the shaken mixture was treated with one volume of deionized water, and the vials were shaken again for 15 min and left for full phase partition. Lastly, the upper layer of PE was diluted, and LYC absorbance was noted at 503 nm. The content of LYP was calculated as µg/g according to the equation written [7].

#### 2.8.3. Determination of Non-Enzymatic Antioxidants

Ascorbic acid (AA) and phenolic content (TPs) of tissue homogenates (3 g) were separately extracted overnight by oxalic acid [10 mL, 4% w/v)] or methanol [25 mL, 80% (v/v)], respectively, followed by centrifugation for each one at 5000× *g* for 20 min at room temperature. Ascorbic acid was quantitively measured as mg g^−1^ by titration of a known volume of the first filtrate against DCPIP dye solution [4]. A known volume of methanolic supernatant or gallic acid gradient concentrations was added to an equal volume of Folin–Ciocalteu reagent in a test tube followed by the addition of 5-fold and 2-fold volumes of sodium carbonate (2N) and distilled water, respectively [4]. Then, samples were kept at 25 °C for 1.5 h followed by reading their absorbance at 760 nm. A standard curve was plotted and utilized to calculate the TP content, expressed as micrograms of gallic acid equivalent per gram of fresh weight.

### 2.9. Lipid Peroxidation

Lipid peroxidation was assessed as micromoles of malondialdehyde (MDA) per gram of fresh weight according to Zhang et al. [42]. MDA was initially extracted from tissue homogenate by 10% (w/v) trichloroacetic acid solution. Centrifugation was then carried out, followed by the addition of thiobarbituric acid solution to a known volume of supernatant. Then, the reaction mixture was boiled for 1 h followed by centrifugation. The clear supernatant was lastly measured at 532 nm.

### 2.10. Activity of Antioxidant Enzymes

Extraction and determination of peroxidase (POD) and catalase (CAT) antioxidant enzymes were, respectively, carried out according to the details documented by [43,44]. One gram of tissue was homogenized in 5 mL sodium phosphate buffer (SPB, 100 mM, pH 6.4) holding 0.1 g PVPP. Centrifugation was then carried out at 12,000× *g* for half an hour at 4 °C and the supernatant was considered for POD enzyme extract. For CAT assay, 2.0 g of frozen fruit tissues were homogenized in 10 mL SPB (50 mM, pH 7.8) at 4 °C. Then, centrifugation was performed under the previous conditions, and the supernatant was used to determine the CAT activity. Protein concentration in enzyme extracts was determined according to the Lowry assay [45] with the help of the bovine serum albumin (BSA) standard curve.

For POD activity, 0.2 mL of crude enzyme extract was added to 2 mL of 100 mM SPB (pH 6.4) and 2 mL of 8 mM guaiacol. After 10 min incubation period, 1 mL of 24 mM H_2_O_2_ was added to initiate the POD reaction. The rise in absorbance at 460 nm was noted using a spectrophotometer (UV-1750, Shimadzu, Kyoto, Japan). In the CAT assay, 500 μL of corresponding enzyme extract was added into a test tube containing 2 mL 50 mM phosphate buffer and 500 μL H_2_O_2_ (12 mM). One unit of POD and CAT activity was defined as the change in OD min^−1^ mg^−1^ protein at 460 and 240 nm, respectively.

### 2.11. Activity of Browning Enzymes

Extraction and determination of polyphenol oxidase (PPO) and phenylalanine ammonia-lyase (PAL) browning enzymes were carried out according to the details documented by Elsherbiny et al. [43] and Assis et al. [46], respectively. The previous POD enzyme extract was also used in the estimation of PPO activity. PAL crude extract was obtained by homogenizing 2 g tomato tissue with 10 mL boric acid buffer (200 mM, pH 8.8) comprising 10% (w/v) polyvinylpyrrolidone, 1 mM EDTA and 50 mM β-mercaptoethanol. Then, centrifugation was performed for 30 min at 10,000× *g* and the resultant clear supernatant was kept under −20 °C for the subsequent assessment of PAL activity.

For PPO activity, 0.5 mL 5% (w/v) catechol was added into a test tube holding 0.2 mL of the crude extract of the enzyme and 1 mL SPB (0.05 M, pH 7.0). After 2 min incubation period, the rise in absorbance at 398 nm was repeatedly noted for 3 min. One unit of PPO activity was defined as a change of absorption in OD 398 min^−1^ mg^−1^ protein. On the other hand, PAL extract (500 μL) was added into a test tube holding 1.5 mL extracting buffer, followed by the addition of 1.5 mL of 20 mM phenylalanine as substrate. To initiate the enzymatic reaction, tubes were then incubated for 3 min at 25 °C, followed by adding 0.1 mL 6 M HCl to terminate the reaction. Finally, absorbance at 290 nm was noted. One unit of PAL activity was defined as a change of absorbance min^−1^ mg^−1^ protein.

### 2.12. Statistical Analysis

All experimental data were subjected to a one-way analysis of variance (ANOVA) in SAS (version 9.1, SAS Institute, Cary, NC, USA). The significant differences between values were considered using Turkey’s HSD test at *p* < 0.05.

## 3. Results and Discussion

### 3.1. Fourier-Transform Infrared Analysis (FTIR)

**Figure 1:**
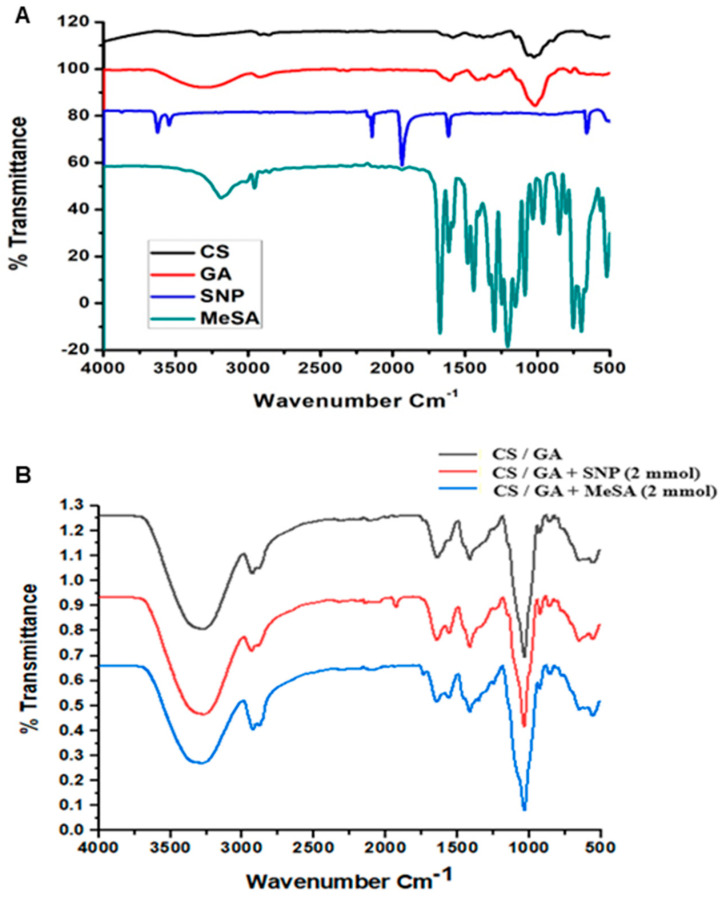
FTIR spectra (**A**) of chitosan (CS), Gum Arabic (GA), sodium nitroprusside (SNP), and methyl salicylic acid (MeSA), and their blends (**B**) of CS/GA, CS/GA/SNP at 2 mM, and CS/GA/MeSA at 2 mM.

Figure 1A,B displays the FTIR spectra of CS, GA, SNP, and MeSA pure powders along with the composite formulations of CS/GA, CS/GA-SNP 2 mM, and CS/GA-MeSA 2 mM. The spectra present characteristic band vibrations of functional groups of each single compound or those yielded in each blend. The IR spectrum of Cs offered the characteristic peaks of 3555, 2863, 1594, 1372, and 1024 cm^−1^. Meanwhile, the peaks at 3284, 2932, 1604, 1410, 1016, and 783 cm^−1^ were noted for GA. The peaks of 3555 in Cs and 3284 cm^−1^ in GA are ascribed to OH stretching groups of the carbohydrate structure. The major IR bands detected at 2863 cm^−1^ in Cs and 2924 cm^−1^ in GA are assigned to the vibrational modes of C–H groups. The characteristic peaks of Cs at 1594 and 1372 cm^−1^ clarify the presence of N–H bending and C–N stretching or amide II [47,48]. The presence of uronic acid carboxylates in the GA structure shows a typical OH in-plane bending band at 1604 and 1410 cm^−1^; a similar finding has been noted [49]. Bands detected in the spectra at 1016 and 783 cm^−1^ may be related to the GA arabinogalactan structure and the linkages of 1–4 and 1–6, respectively, for galactose and mannose moieties in GA [50]. Overall, previous findings related to the Cs and GA FT-IR spectra in this work were in harmony with those formerly documented [7,14,49,50].

The strong absorption centered at 2141 cm^−1^ in the spectrum of SNP is assigned to the C≡N stretching vibration [51]. The very strong band at 1935 cm^−1^ is assigned to the NO stretching vibration [52]. The absorption band at 669 cm^−1^ is assigned to the vibration involving the Fe–N–O grouping [51]. The FT-IR spectrum of SNP largely agreed with those previously reported [52,53]. The spectrum of MeSA displays notable absorption bands at 3186 and 2958 cm^−1^ for the O–H stretching vibration and C–H stretching, respectively. While the characteristic peak of the ester C=O group was noted at 1674 cm^−1^. The peaks appeared between 1613–1441 cm^−1^ in MeSA assigned to the C=C stretching vibrations. The strong bands between 1152–1031 cm^−1^ indicate C-O stretching due to the phenolic OH group. The peak at 669 cm^−1^ recognized the presence of the hydroxyl group out-of-plane bending of the bounded OH group, peaks appeared at 849 and 695 cm^−1^ indicating the presence of an out-of-plane aromatic ring bend [54]. The FTIR spectrum of MeSA was in harmony with those formerly obtained [28,55].

As a result of the interactions between Cs and GA, the carbonyl-amide region was meaningfully altered so that the bending vibration of N–H was shifted from 1594 to a weaker peak at 1555 cm^−1^ (Figure 1B), while the weak intensity of the 1372 cm^−1^ absorption peak of amide II disappeared. Moreover, the absorbance peak of 1410 cm^−1^ of GA was slightly shifted into 1414 cm ^−1^ in the CS/GA blend. This could be caused by the electrostatic interaction between the negative functional groups of GA (COO^−^) and the positive ones (NH_3_^+^) of CS [56]. The change in the FTIR spectrum for SNP dissolved in a reduced volume of water and quickly introduced into a CS/GA mixture solution after photoirradiation is shown in Figure 1B. The absorbance peak of 2141 cm^−1^ assigned to C≡N in SNP became extremely weaker in the CS/GA-SNP composite. Moreover, the strong peak found at 1935 cm^−1^ related to the NO stretching vibration in SNP was shifted to a weaker intensity peak at 1925 cm^−1^ in the CS/GA-SNP composite. The clear reduction in the vibrational peak of CN and NO groups in CS/GA-SNP indicates SNP dissociation to some extent. Similarly, Liu et al. [52] found an extreme decline in the vibrational peak of the aforementioned two functional groups in the SNP aqueous solution irradiated for 20 min. The peak that appeared at 648 cm^−1^ related to the CS/GA backbone seemed to be broader at the same wavelength in CS/GA-SNP due to the interference with that of the Fe–N–O grouping in non-dissociated SNP. The peak ascribed to OH groups of the carbohydrate structure in CS/GA observed at 3280 cm^−1^ was shifted to a broader one at 3268 cm^−1^ in the CS/GA-SNP composite.

**Figure 2:**
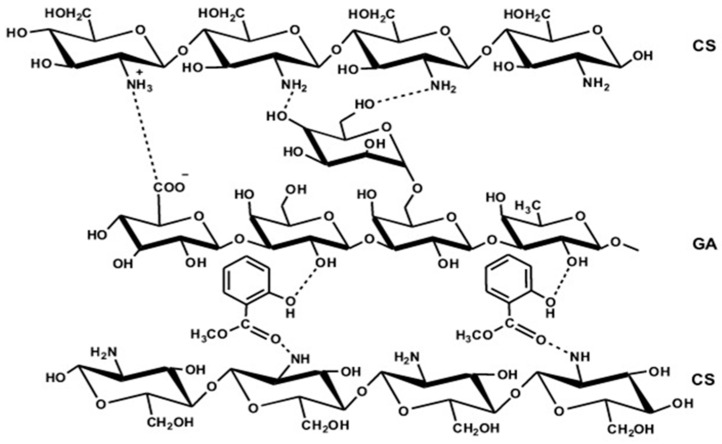
The proposed structure of the CS/GA-MeSA composite.

The absorption bands at 3280 and 2927 cm^−1^ in the CS/GA blend were slightly shifted to other frequencies (3292 and 2924 cm^−1^) for Cs/GA-MeSA 2 mM as shown in Figure 1B due to the formation of hydrogen bonds between the functional groups of MeSA particles with the CS/GA-blend functional groups, particularly OH groups; a similar observation has been documented by Lee et al. [28]. They reported a slight shifting to lower or higher frequencies in the peaks related to the β-cyclodextrin (β-CD) hydroxyl groups in the MeSA/β-CD different blends. They also showed that all MeSA/β-CD different blends had spectra mostly different than MeSA. Similarly, the spectrum of the CS/GA-MeSA 2 mM film in the present work presented a loss of the characteristic peak zones related to MeSA (1152–1031 cm^−1^ and 1674–1441 cm^−1^). This could be explained by the presence of strong peaks at corresponding frequency ranges (1031, 1414, and 1555 cm^−1^) related to the carbohydrate backbone of the CS/GA blend. Likewise, the probability of hydrogen bonding with the MeSA C=O group might shift its peak (1674 cm^−1^) to a slightly higher wavenumber of 1735 cm^−1^ in Cs/GA-MeSA 2 mM, elucidating the entrapping of MeSA in the CS/GA blend. Overall, the appearance or absence of an MeSA carbonyl peak in the IR spectrum is affected by the solvent type [54]. Overall, our preliminary studies presented that the increase in MeSA concentration (1–10 mM) in the CS/GA blend caused more a profound frequency shifting or disappearance of OH groups regarding CS/GA to lower wavenumbers accompanied by the disappearance of other peaks related to the carbohydrate backbone due to the strong attendance of MeSA frequencies; similar observations have been noted [57]. Figure 2 displays the proposed structure of the CS/GA-MeSA composite.

### 3.2. In Vitro Release of NO from CS/GA-SNP Blends

**Figure 3:**
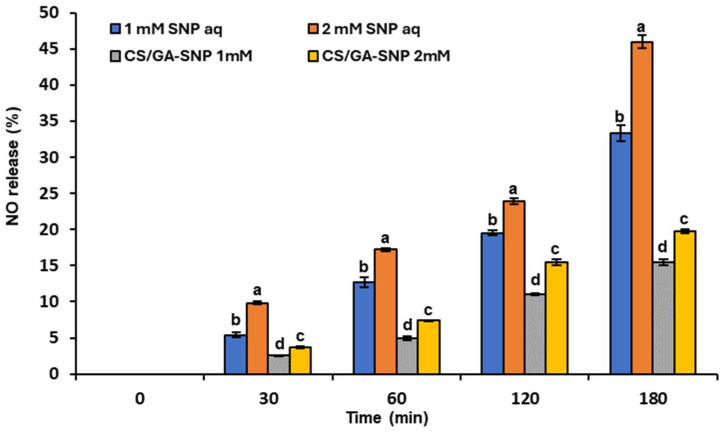
Temporal kinetics of NO release from free aqueous sodium nitroprusside (SNP) or incorporated in CS/GA solution blend at the concentrations of 1 and 2 mM. Results represent the mean ± standard error (SE). Bars with different letters are significantly different according to Tukey’s HSD test at *p* < 0.05 for the same time point.

Recently, the encapsulation of NO donors is a promising strategy to allow a more controlled release and increase their protection from accelerated degradation. In this respect, the data shown in Figure 3 indicate the percentages of NO released by 1- and 2-mM SNP separately added into distilled water or CS/GA blends. The release rate of NO from CS/GA-SNP 1 mM and CS/GA-SNP 2 mM were significantly lower than its release in distilled water. Supposedly, the complete dissociation of SNP (100%) led to the formation of NO in concentrations of 1 and 2 mmol L^−1^. At 180 min, the percentages of NO released from CS/GA-SNP 1 mM and CS/GA-SNP 2 Mm were only 15.46 and 19.73 of the theoretical value, whereas the percentages of NO released from the 1 and 2 mM SNP aqueous solutions were 33.33 and 46 of the theoretical value. The findings clarify that CS/GA significantly reduced the NO release rate in the earlier tested solutions; a similar result has been described by Ma et al. [39]. They found that the NO release from nitrosoglutathione in CS nanoparticles was significantly lower than in CS. Likewise, Silveira et al. [58] found that the release of NO from SNP was significantly lower than those of other NO donors like S-nitroso-mercaptosuccinic acid, S-nitrosoglutathione, or S-nitroso-N-acetylcysteine when encapsulated in CS during 24 h.

### 3.3. Antifungal Activity

**Figure 4:**
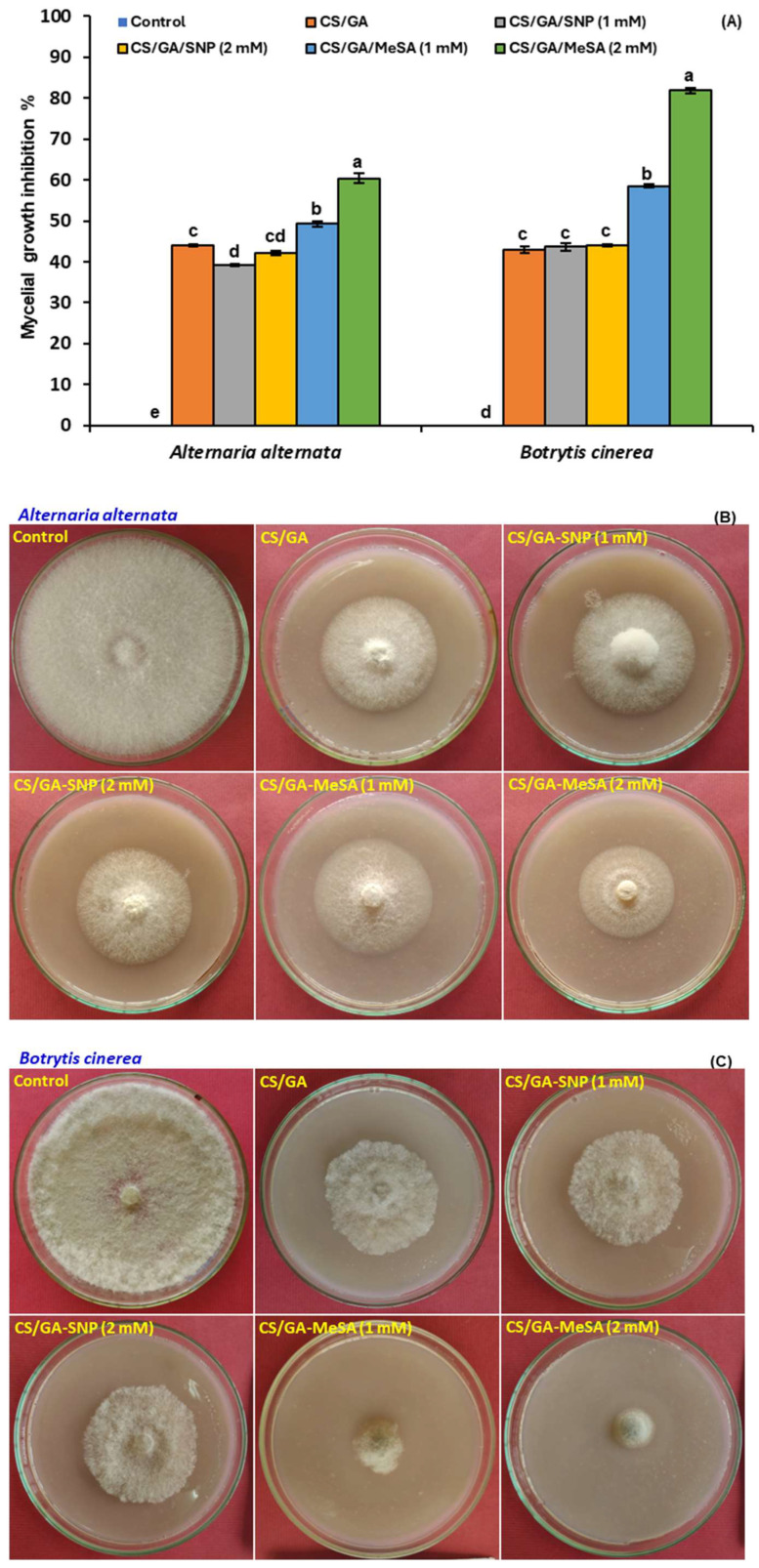
Effect of CS/GA, CS/GA-SNP (1 and 2 mM), and CS/GA-MeSA (1 and 2 mM) composites on (**A**) mycelial growth inhibition (%) and radial growth of *Alternaria alternata* (**B**) and *Botrytis cinerea* (**C**). Results represent the mean ± standard error (SE). Bars with different letters are significantly different according to Tukey’s HSD test at *p* < 0.05.

The antifungal potential of CS/GA, CS/GA-SNP 1 mM, CS/GA-SNP 2 mM, CS/GA-MeSA 1 mM, and CS/GA-MeSA 2 mM formulations against *A. alternata* and *B. cinerea* was assessed using the radial growth method. Interestingly, the mycelial growth of both fungal pathogens was significantly reduced in all studied polymer mixtures on the fifth day of incubation in comparison to the control (Figure 4A–C). *A. alternata*, CS/GA, CS/GA-SNP 1 mM, CS/GA-SNP 2 mM, CS/GA-MeSA 1 mM, and CS/GA-MeSA 2 mM formulations exhibited inhibition zones of 44.1, 39.2, 42.2, 49.2, and 60.3%, respectively. The previous formulations produced an extensive inhibition in *B. cinerea* mycelial growth by 42.9, 43.7, 44.1, 58.5, and 81.8%, respectively. Remarkably, MeSA significantly improved the antifungal activity of the CS/GA mixture against *A. alternata* and *B. cinerea*.

The antifungal action of the CS/GA blend is principally based on the ability of free positive moieties of CS in the polymer blend to attract electrostatically with those negatively charged in fungal cell membrane phospholipids, causing their disruption [59]. After that, CS can pass through the cell and inhibit the synthesis of nucleic acids. The enhancement of the previous blend with SNP did not improve the antifungal activity in this study. Similarly, it was recently detected by Zhang et al. [60] that the mycelial growth of *A. alternata* was not inhibited by the application of SA or SNP when tested in vitro. However, the biofilm inhibition activity of silica nanoparticles holding SNP as an NO donor agent against *Staphylococcus* strains has been reported [61].

Numerous documents have stated the antimicrobial properties of MeSA [62,63]. Contrarily, Lin, et al. [64] found the opposite effect of MeSA on *Lecanicillium lecanii*, where it improves both fungal growth and toxicity. In the present study, incorporating MeSA into the CS/GA blend significantly improves its antifungal activity. To our knowledge, this is the first document to evaluate the antifungal activity of the CS/GA blend alone or enriched by SNP or MeSA against *A. alternata* and *B. cinerea*, principally affecting the quality of postharvest tomato fruits.

### 3.4. Transmission Electron Microscope (TEM)

**Figure 5:**
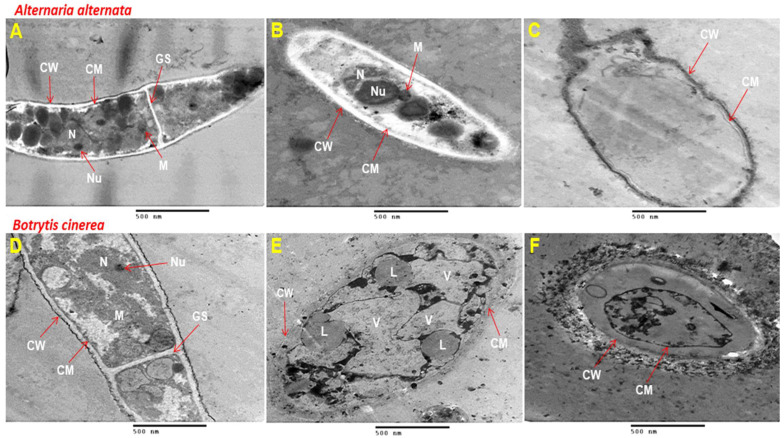
Transmission electron microscopy (TEM) micrographs of *Alternaria alternata* and *Botrytis cinerea* treated with CS/GA-MeSA at 2 mM: (**A**,**D**) untreated hyphae, and (**B**,**C**,**E**,**F**) treated hyphae. Cell wall (CW), cell membrane (CM), nucleus (N), nucleolus (Nu), mitochondria (M), vacuole (V), lipid droplets (L), and germination septae (GS).

TEM observations for untreated mycelium of *A. alternata* showed normal hyphae with abundant cytoplasmic organelles, and a continuous cell wall with no cracks seen along the cell membrane (Figure 5A). By contrast, the treated hyphae with MeSA in the CS/GA mixture at 2 mM exhibited obvious sub-cellular structural perturbations, shrinkage, and altered germination septae (GS). Amazingly, *A. alternate* exposed to the treatment of CS/GA-MeSA at 2 mM represented severe findings—typically, corroded and cracked cell walls (Figure 5B), sometimes with no cytoplasmic organelles (Figure 5C).

The untreated mycelium of *B. cinerea* exhibited a typical ultrastructure, specifically, cytoplasm, mitochondria (M), germination septae (GS), nuclei’s distinct envelopes (N), and clear nucleolus (Nu). Furthermore, the outside cell wall (CW) and inner cell membrane (CM) were flawless and well-structured (Figure 5D). On the contrary, the treated fungal culture with CS/GA-MeSA at 2 mM led to the formation of enormous vacuoles (V) and lipid droplets (L) (Figure 5E). Moreover, there were the shrinking of the cytoplasmic contents and the separation of the cell membrane (CM) from the cell wall (CW) with the complete deformation of the cell (Figure 5F).

To date, there is no scientific literature about the effect of the mixture of CS/GA-MeSA on the ultrastructure of *A. alternata* and *B. cinerea*. However, many reports are available about the alterations in the cell ultrastructure for both fungi using natural and chemical treatments. In this context, the 1% *Litsea cubeba* essential oil caused conspicuous alterations of *B. cinerea* mycelia including the destruction of the cell wall, ruptured cell membrane, leakage of cytoplasmic components, and loss of the normal shape of the mycelia [65]. Similarly, the mycelia of *B. cinerea* grown in the presence of cembratrien (CBT)-diols are carbocyclic diterpenes, isolated from the inflorescence of the flue-cured tobacco, and, at 200 μg/mL, showed many of the abnormal features that are cytological and ultrastructural. This treatment destroyed the internal tissue structure and damaged the structure of the cell wall and membrane system, inducing the leakage of cellular materials [66]. Moreover, at the concentration of 200 mg/L from ferulic acid (FA), methyl ferulate (MF), and ethyl ferulate (EF), the internal structure of *A. alternata* was more seriously damaged. The cytoplasm was condensed into fragmentary shapes, and some organelles were dissolved gradually in all treated mycelia [67].

### 3.5. Physiochemical Changes during Shelf Life

#### 3.5.1. Fruit Firmness

**Figure 6:**
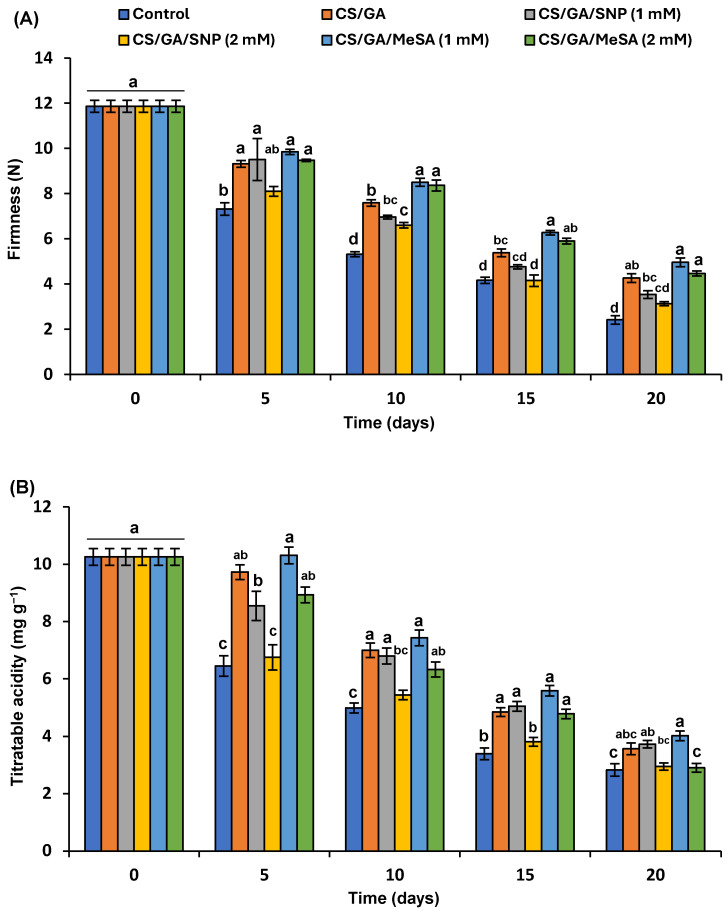

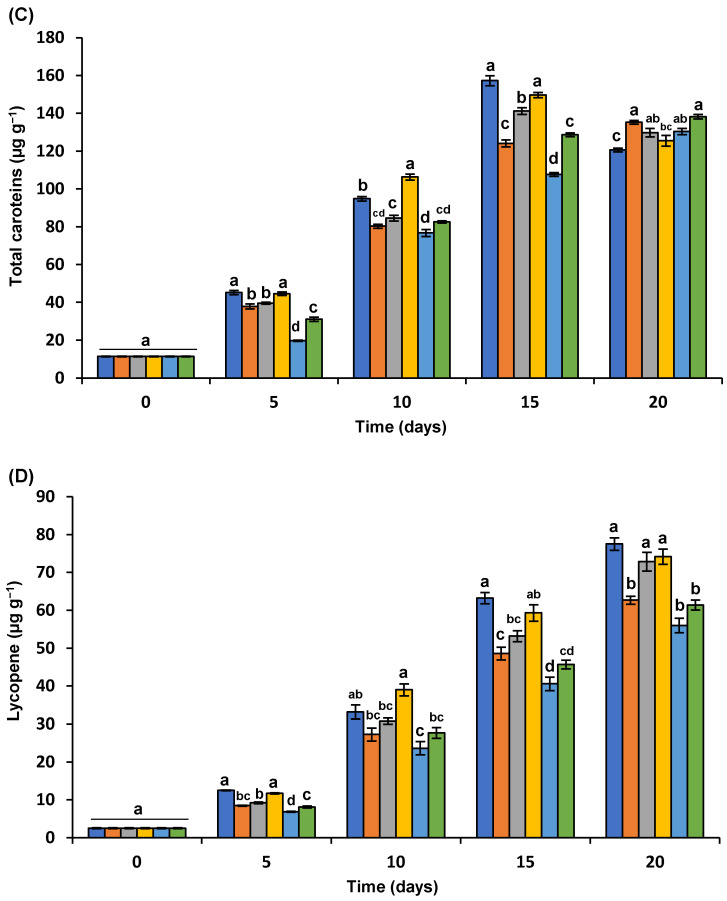
Impact of coating composites on (**A**) firmness, (**B**) titratable acidity, (**C**) total carotenoids, and (**D**) lycopene in tomatoes during the shelf-life period. Results represent the mean ± standard error (SE). Bars with different letters are significantly different according to Tukey’s HSD test at *p* < 0.05 for the same time point.

In the present study, the firmness of tomato fruits was significantly reduced in all tested samples until the entire ripening period (Figure 6A). It could be noticed that the control uncoated fruits had the lowest significant levels of firmness throughout the experiment period. The application of CS/GA-MeSA 1 mM, CS/GA-MeSA 2 mM, and CS/GA treatments had higher significant levels of firmness in comparison to the control throughout the experiment; the previous groups had values of 4.9, 4.4, 4.2, and 2.4 N at the 20th day of storage (Figure 6A). Tomato fruit loses its firmness due to the rapid development of ripening reactions, therefore diminishing its shelf-life period [68]. The high activity of cell-wall-degrading enzymes resulted in fruit softening [7]. During softness, pectinesterase and hemicellulolytic enzymes depolymerize pectin constituents and hemicelluloses, respectively, and, therefore, cause cell wall disruption [7,68]. Therefore, the rapid drop of firmness values in the control group could be clarified by the impact of hydrolytic enzymes which degrade the cell wall, causing fruit softening. In the last decade, biodegradable coatings have been used extensively to support sustaining the firmness of different fruits by decreasing the respiration rate, decelerating ripening, hindering the degradation of cell walls, and delaying senescence. The presence of MeSA in the CS/GA blend succeeded in sustaining a higher level of firmness in tomato fruit throughout the storage period. Moreover, improving the efficacy of edible coatings to avoid fruit softness through their blending with different chemical elicitors like INA and SA has been described [7,40]. In this regard, Giménez et al. [69] reported that MeSA vapors effectively maintain the quality of postharvested sweet cherries during storage via a decreasing respiration rate, weight loss, softening, and total acidity. As far as we know, no earlier reports have evaluated the role of MeSA in polymer-coating films to delay the ripening of postharvest tomato fruits. However, the ripening retardation of tomato fruit by MeSA vapors via the down-regulation of ethylene biosynthetic genes has been reported [70]. In a different technique, Cholmaitri et al. [57] also found that the controlled release of MeSA by some biosorbents conserves the firmness and retards the ripening of banana fruit via the inhibition of ethylene production.

#### 3.5.2. Titratable Acidity

The TA of all tested fruits in this study decreased after ten days until the termination time of storage. It could be noticed from (Figure 6B) that uncoated fruits and CS/GA-SNP 2 mM groups possessed a sharp decrease in TA contents from the fifth day of storage until the end of storage. Contrarily, a low level of increase in TA happened on the fifth day by the CS/GA-MeSA 1 mM formulation followed by a slow decline until the termination time of the experiment. The TA percentages of the aforementioned groups, i.e., the control, CS/GA-SNP 2 mM, and CS/GA-MeSA 1 mM formulation, on the 20th day of storage were 2.8, 2.9, and 4.0, respectively (Figure 6B).

The TA increase after five days of storage for the CS/GA-MeSA 1 mM formulation is generally attributed to the higher level of accumulated organic acids (OAs), while the subsequent decrease with different scores in all tested groups could be attributed to the development of ripening processes where OAs could be used as a substrate in sugar metabolism or expanded in respiration reactions [71]. Overall, our study suggests that the CS/GA-MeSA 1 mM formulation is the best treatment for inhibiting respiration and, therefore, reduces the use of OAs during the shelf-life period, and, accordingly, results higher acidity contents. Similarly, Zhu et al. [12] stated that the acidity reduction was lower in nano-silicon oxide/chitosan-treated tomato fruits than in uncoated fruits. Moreover, the retarding effect of the Cu-chitosan nano-net film on tomato fruit respiration and preserving higher TA values has been previously stated [72]. Recently, Taher and Elsherbiny [7] found that the CS/PVA blend enriched by INA could retard ripening and conserve higher TA values.

Principally, the present work exhibited that the CS/GA-MeSA 1 mM formulation had the highest significant contents of TA throughout the trial period, indicating that MeSA in a concentration of 1 mM in the polymer blend has a possible role in extending the tomato fruit shelf life. In this regard, the inhibitory action of 0.1 mM MeSA vapors on ethylene biosynthesis has been reported in tomato fruits [70]. They also showed that the higher concentration of 0.5 mM MeSA vapors was able to inhibit red color development. Moreover, CS was able to inhibit the respiratory degree and the breakdown of chlorophyll [7]. Interestingly, 1 mM MeSA in the CS/GA blend was able to decrease ripening with an adequate rate of ethylene production without any suppression in red color development.

In the case of SNP, its preharvest aqueous treatments sustained firmness and caused higher TA values than those of the control in postharvested tomatoes during cold storage [73]. Likewise, NO released from the 1 mM SNP aqueous solution was able to retard fruit maturity via decreasing ethylene production, and, therefore, significantly elevated the firmness and TA compared to the control [32]. Contrarily, our results showed that NO released by SNP in the CS/GA blend had no impact on maturity retardation, particularly by using the CS/GA-SNP 2 mM formulation where it decreased the firmness and TA to low levels. Overall, the data obtained from Figure 3 elucidated that NO release in the CS/GA polymer blend was largely lower than that in aqueous solutions.

#### 3.5.3. Total Carotenoids

Figure 6C displays the pattern of the TC level of tomato fruits treated with different formulations based on CS/GA during 20 days of storage. The TC content gradually elevated in the control- and CS/GA-SNP 2 mM-treated fruits and reached the highest significant values for the aforementioned groups as 157.2 and 149.7 µg/g F.W, respectively, after the fifteenth day, followed by a noteworthy decrease on the 20th day of storage. Remarkably, the formulation of CS/GA-MeSA 1 mM showed a slower level of increase in TCs where it achieved the lowest significant values of 19.77, 76.73, and 107.7 µg/g F.W after 5, 10, and 15 days, respectively.

The biogenesis of cyclic carotenoids is enzymatically induced during fruit maturity. The maximum rate of cyclization enzyme activity was described to be in the green period of tomatoes [74], whereas the maximum content of TCs has been documented to occur in ripe fruits [7,74]. In this study, the TC content was raised regularly during the fifteenth day of storage followed by a massive drop in control, the CS/GA-SNP 2 and CS/GA-SNP 1 mM groups reflected their earlier maturity than those immersed in other formulations as well as the weakness of SNP in CS/GA to slow maturity. Similar results have been described concerning changes in TC levels in untreated fruits [4]. The substantial decrease in TCs in fruits immersed in CS/GA when compared to untreated fruits on the 10th and 15th days of storage reflects the efficiency of the CS/GA mixture in delaying ripening to some degree. This result mostly agreed with that formerly recorded by Taher and Elsherbiny [7], who noted that CS/PVA decreased the maturation rate by reducing the respiration extent and, therefore, accumulated higher levels of TCs at the late ripening stage than untreated fruits. The minor continuous rise in TC content of CS/GA-MeSA 1 mM until the 20th day of the experimental time in this work illustrates the prospective function of MeSA in retarding the ripening reactions. A similar observation has been recently reported by Taher and Elsherbiny [7], who found that isonicotinic acid in CS/PVA as an elicitor resulted in a slow level of increase in TCs. To our knowledge, no papers have been documented on the influence of MeSA in polymer-coating films on the development of common pigments in tomato fruit.

#### 3.5.4. Lycopene Content

Lycopene levels were elevated during the shelf-life period in all coated and uncoated fruits (Figure 6D). The lycopene content was mildly elevated in all examined samples until the fifth day; then, a sharp increase with significance in uncoated fruits and the CS/GA-SNP 2 mM formulation happened until the entire period of storage. It reached the maximum values of 77.5 and 74.1 µg/g F.W for control samples and the CS/GA-SNP 2 mM-treated fruits on the 20th day of the shelf-life period. Contrarily, a mild continued increase with significance in the lycopene amount was noticed in CS/GA-MeSA 1 mM-treated fruits until the full time of the trial (Figure 6D); it had the lowest significant content on the 20th day of storage as 55.9 µg/g F.W.

In this study, the slow pigmentation rate in tomato fruits treated by the CS/GA blend supplemented with MeSA reflects the delay in ripening reactions, which might be due to the reduction in ethylene biosynthesis. Interestingly, no earlier studies have assessed the effects of MeSA in polymer-coating films on the physicochemical characteristics of fruits during the shelf-life period. However, the inhibitory effect of MeSA vapor treatment on ethylene production has been reported in postharvest sweet cherry fruits [69]. The dual regulatory impact of MeSA vapor on gene expression related to the biosynthetic pathway of ethylene in tomato fruit has been previously noted [70]. They concluded that a high concentration of MeSA vapor (0.5 mM) inhibited lycopene accumulation, respiration, and ethylene biosynthesis during ripening. The lower concentrations enhance ripening in green or breaker-stage tomatoes.

In the case of SNP, its preharvest aqueous treatments at a concentration of 200 μM sustained a higher lycopene content than that of the control in postharvested tomatoes during cold storage [73], while the aqueous solution of SNP 1 mM was able to retard the maturity of tomato fruits inoculated by *B. cinerea* via decreasing ethylene production and, therefore, significantly conserve firmness and TA [32]. Contrarily, SNP in the CS/GA blend in the present study significantly reduced firmness and TA simultaneously with a higher lycopene content resembling uncoated fruits, and, thus, this reflects the weakness of the triple composite, i.e., CS/GA-SNP on ethylene production and ripening reactions.

#### 3.5.5. Ascorbic Acid Content

**Figure 7:**
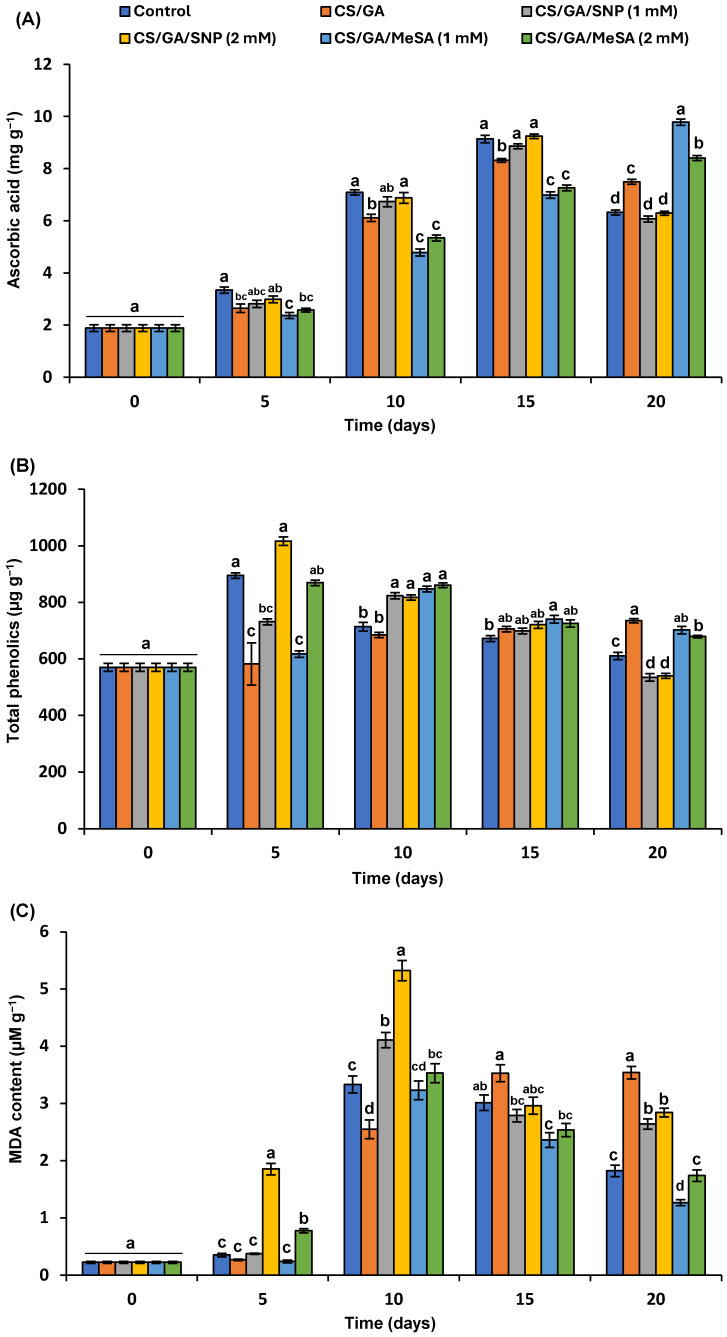
Effect of each blend treatment on (**A**) ascorbic acid, (**B**) total phenolic, and (**C**) malondialdehyde (MDA) contents in tomatoes during the shelf-life period. Results represent the mean ± standard error (SE). Bars with different letters are significantly different according to Tukey’s HSD test at *p* < 0.05 for the same time point.

The level of ascorbic acid (AA) is progressively increased until the full maturity of tomato fruit, followed by a significant reduction [4]. The AA content gradually elevated in the control, CS/GA-SNP 1 mM, and CS/GA-SNP 2 mM groups where they reached the highest values of 9.1, 8.8, and 9.2 mg/g on the 15th day, and then notably decreased to lower values indicating the rapid maturation of the aforementioned groups (Figure 7A). The fruits treated with CS/GA-MeSA 1 mM and CS/GA-MeSA 2 mM had lower significant values of AA against control until the 15th day, before being much significantly higher at the complete period of storage with the values of 9.78 and 8.40 mg/g FW, respectively. The previous results display that all CS/GA biodegradable films containing MeSA defeat the decline in ascorbic acid load, referring to the over-maturity of tomatoes. An identical pattern for delaying the decline in AA in tomato fruits by edible polymers has been stated [7,68,75].

It is well-known that edible coatings supplemented with natural antioxidants have a preservative impact on AA and preserve it with higher content in tomato fruits [4,76,77]. The antioxidant activity as a free radical scavenger of MeSA-dominated essential oils has been reported [78]. However, it appears that no immediately obtainable information exists regarding the influence of MeSA on AA. Noticeably, the significantly higher contents of ascorbic acid on the 20th day of the experiment in the CS/GA-MeSA 1 mM and CS/GA-MeSA 2 mM groups (Figure 7A) could be attributed to the defensive function of MeSA on the ascorbic acid load, alongside maturity delay.

#### 3.5.6. Total Polyphenols

The TP quantity in the CS/GA-SNP 2 mM and control groups recorded the maximum peak on the fifth day as 1016.98 and 895.2 µg GAE/g, respectively, while CS/GA-SNP 1 mM, CS/GA-MeSA 1 mM, and CS/GA-MeSA 2 mM reached the highest peak on the 10th day with values of 824, 847.46, and 860.83 µg GAE/g, respectively, and then slowly decreased on the 20th day (Figure 7B). In Cs/GA-treated fruits, the TP level increased slowly and reached the highest value (735.39 µg GAE/g) at the end of the storage period. Remarkably, the CS/GA-SNP 2 mM and CS/GA-SNP 1 mM groups exposed the lowest significant TP values by the entire shelf-life period.

Chitosan coating improved the quality features of different fruits by declining the gas exchange and moisture dropping and evading postharvest fungal pathogens [40]. TPs are extensively accumulated in plant tissues as a defensive response against biotic/abiotic stress conditions like fungal attack [79]. In the current study, the CS/GA film demonstrated lower total polyphenol (TP) levels compared to the control until day 10. This finding is consistent with Zhu et al. [12], who observed that both CS and nano-SiOx/CS films decreased the TP content in tomatoes compared to that of uncoated tomatoes. Interestingly, our study showed that CS/GA blended with 1 mM MeSA caused a significant rise in TP content, predominantly in the late stage of storage. The previous finding generally disagreed with that formerly noted by Taher and Elsherbiny [7], who found that CS/PVA biopolymer incorporated with isonicotinic acid (INA) significantly decreased TP values compared to untreated breaker fruits. Contrarily, SNP treatment has been documented to accumulate huge levels of TPs and TFs in different fruits and vegetables throughout the storage period [80]. However, in this study, SNP in CS/GA accumulate huge levels of TPs at the early phase of storage, followed by demonstrating a huge decline in the late period of storage. Interestingly, no earlier papers have assessed the influence of SNP or MeSA incorporated in edible coatings on the accumulation of TPs in tomato fruits during storage.

#### 3.5.7. Malondialdehyde (MDA)

MDA is a secondary metabolite resulting from lipid peroxidation induced by ROS. Therefore, its abnormal level due to the cell membrane damage is directly proportional to the development of oxidative stress. Figure 7C shows the impact of CS/GA treatments on the level of lipid peroxidation weighed as µmoles of MDA per gram tissue. Its content largely increased at the 10th day of postharvest storage in all tested samples, followed by a slow decrease in some treatments in the late stage of storage (Figure 7C). CS/GA-SNP 2 mM treatment showed the maximum significant value of MDA on the fifth day of storage as 1.853 µmoles of MDA per gram FW; the same group also reached the maximum peak at the 10th day with the value of 5.324 U, while CS/GA-MeSA 1 mM treatment displayed the lowest significant values after 15 and 20 days as 2.36 and 1.26 µmoles of MDA per gram FW, respectively. In other words, the CS/GA-MeSA 1 mM coating treatment had significantly (*p* < 0.05) lower contents of MDA than that of the control group from the 15th day until the termination time of the experiment; similar findings have been reported [79]. They found that CS/PVP biopolymer incorporated with salicylic acid can decrease the MDA level of Banati Gauva fruits under low temperatures and, therefore, decrease oxidative damage. Moreover, the spraying of ‘Sucrier’ bananas with an MeSA solution during storage has been reported to activate the ascorbate-glutathione cycle and, therefore, eliminate ROS and decrease MDA level [81]. The immersing of ripening banana fruit in an MeSA solution during the storage period diminished the H_2_O_2_ load and brown spots [82].

The incorporation of edible coatings with bioactive additives has been documented to maximize the reduction level of MDA during postharvest stages of fruits and, thus, conserves the functions of cellular membranes, as well as diminishes cell permeability levels [7,76]. Our results regarding lipid peroxidation exposed that CS/GA-MeSA 1 mM is a suitable tool for delaying postharvest oxidative damage. This might be explained by the ability of the CS/GA film to generate a barrier to O_2_ liable for lipid peroxidation [83] and the ability of MeSA to face the internal stress of the CS/GA film. Overall, salicylic acid and its derivatives such as MeSA have been documented to reduce fruit senescence during postharvest storage [14]. To our knowledge, no papers have evaluated the effect of MeSA in coating films on the peroxidation reactions of membrane lipids during the shelf life of harvested fruits. Nitric oxide (NO), an important signaling molecule resulting in SNP solutions, can prevent the accumulation of MDA in different fruits and vegetables [80]; however, it had a different pattern when incorporated in CS/GA in this study.

### 3.6. Antioxidant and Browning Enzymes

**Figure 8:**
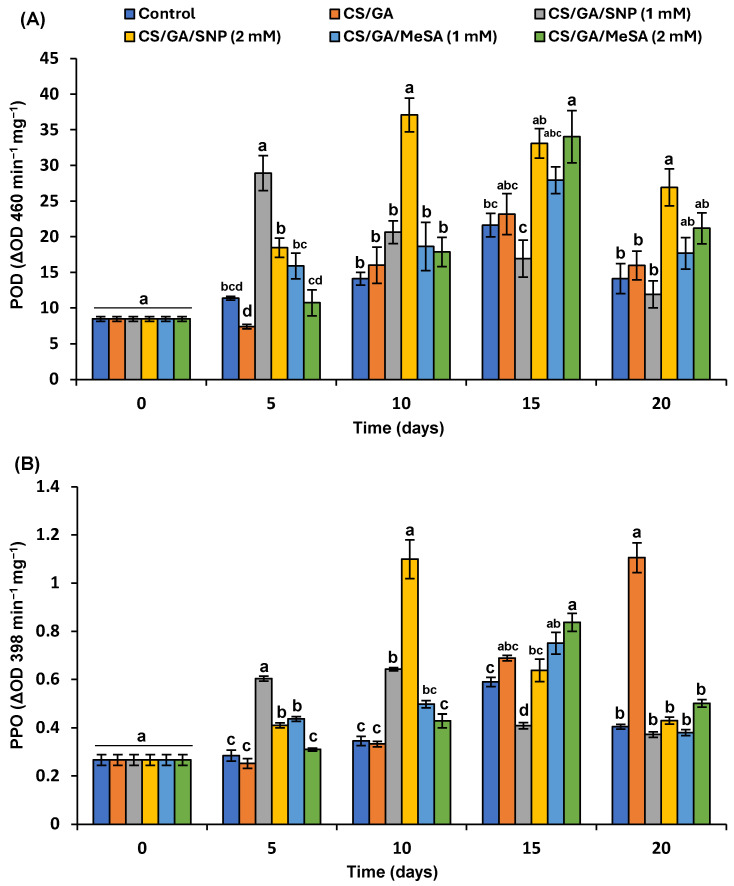

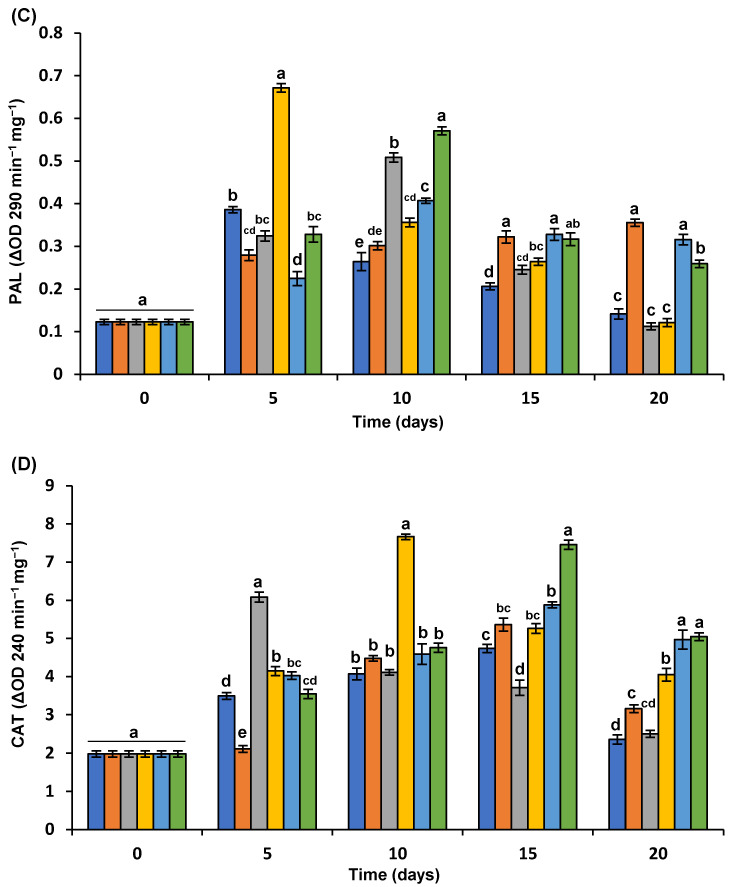
Effect of blend treatments based on CS/GA on enzyme activity of (**A**) peroxidase (POD), (**B**) polyphenoloxidase (PPO), (**C**) phenylalanine ammonia-lyase (PAL), and (**D**) catalase (CAT) in tomato fruit during the shelf-life period. Results display the mean ± standard error (SE). Bars with different letters are significantly different according to Tukey’s HSD test at *p* < 0.05 for the same time point.

In the case of POD, its activity recorded the maximum peak on the fifth day in the CS/GA-SNP 1 mM group (28.9 U); then, a sharp drop occurred until the termination time of storage, while the CS/GA-SNP 2 mM set attained the maximum of the peak on the 10th day (37.0 U) followed by a slow decline in its activity until the termination time of storage (Figure 8A). All other sets achieved the maximum peak on the 15th day where the CS/GA-MeSA 2 mM formulation recorded the highest significant value as 34.0 U. As can be seen, fruits treated with CS/GA-MeSA 1 mM blend had higher but non-significant values of POD activity than those of control untreated fruits throughout the trial.

In the polyphenol oxidase (PPO) assay, CS/GA-SNP 2 mM-treated fruits reached the maximum peak with the highest significant activity (1.1 U) on the 10th day of the shelf-life duration. Meanwhile, the formulation of CS/GA-MeSA 2 mM and CS/GA-MeSA 1 mM displayed the highest significant activity of PPO on the 15th day at 0.83 and 0.75 U, respectively. The fruits treated with the CS/GA blend had lower non-significant values of PPO activity than the untreated group in the early stage of the trial before the incidence of a huge increase at the termination time (Figure 8B).

Figure 8C showed that the PAL activity of the CS/GA-SNP 2 mM-coated fruits recorded the maximum significant value on the fifth day as 0.67 U, and then sharply declined until the termination time of the trial. Remarkably, the CS/GA-MeSA 1 mM and CS/GA treatments had a lower significant activity of PAL than the control uncoated fruits in the early stage of storage (Figure 8C). The application of the CS/GA-MeSA 2 mM, CS/GA-SNP 1 mM, and CS/GA-MeSA 1 mM treatments reached the maximum peak on the 10th day of ripening with significant values of 0.57, 0.50, and 0.40 U, respectively. The fruits treated with the CS/GA blend had a slow but continuous increase in PAL activity, where it had the highest significant value on the 20th day after treatment.

Figure 8D shows that the CAT activity gradually elevated in the early phase of ripening for the CS/GA-SNP 1 mM-treated fruits, attaining the highest peak on the fifth day as 6.083 U, before decreasing. Likewise, CS/GA-SNP 2 mM-treated fruits reached the maximum peak with the highest significant activity (7.6 U) on the 10th day of the experimental time. The formulations of CS/GA-MeSA 2 mM, CS/GA-MeSA 1 mM, CS/GA, and control reached the highest peak of CAT on the 15th day at 7.459, 5.883 5.363, and 4.74 U, respectively.

PAL, POD, PPO, and CAT are defense-related enzymes generally related to the formation of induced plant resistance as opposed to pathogenic fungi [43]. PAL is a crucial enzyme in the phenylpropanoid biosynthetic reactions responsible for the accumulation of defense-related secondary metabolites like lignin, phenols, and flavonoids in plants [84]. PPO is complicated in the plant cell lignification process and contributes to plant polyphenols’ oxidation into related quinones, which are extremely toxic to numerous plant pathogenic fungi [85]. The activity of POD is a critical step in cell wall lignification and phenolic oxidation. The activity of POD is used to indicate the plant resistance grade to pathogenic diseases [86]. The activities of CAT and POD as antioxidant enzymes in plant tissues aim to avoid ROS rising via the dismutation of H_2_O_2_ into H_2_O and O_2_. In the present work, a significant elevation in PAL, PPO, POD, and CAT activities was detected in tomato fruits coated with CS/GA-SNP 2 mM compared with the uncoated set. The improved activity of the previous enzymes in the early or middle stages of ripening by CS/GA-SNP 2 mM will rapidly induce the biosynthesis of different types of antifungal secondary metabolites, particularly lignin, and polyphenols via initiating the phenylpropanoid metabolism pathway, thereby elevating the capability of tomato fruit to defeat fungal diseases. Excitingly, there are no published studies that have evaluated the influence of NO released by SNP in edible polymer solutions on the activity of antioxidant and browning enzymes in postharvest fruits. However, NO application via its release from 1 mM SNP aqueous solution on tomato fruits inoculated by *B. cinerea* has been studied [32]. They found that SNP was able to retard fruit maturity and significantly increase CAT, SOD, and POD activities but in a late period of storage compared to the control, while NO released by SNP in the CS/GA blend in this study significantly increased antioxidant and resistance enzymes but in the early stage of ripening without any impact on ripening retardation. Likewise, preharvest aqueous SNP treatment significantly improved superoxide dismutase and POD enzyme activities than that of the control in postharvest tomatoes during cold storage [73]. Overall, the stimulatory role of NO in elevating the mRNA expression level of antioxidant genes in tomato fruit has been reported [32].

Moreover, the CS/GA-SNP 1 mM, CS/GA-MeSA 1 mM, and CS/GA-MeSA 2 mM formulations were able to induce resistance by activating defense enzymes but in advanced stages of maturation in a different manner obtained by CS/GA-SNP 2 mM. As antioxidant enzymes have been chiefly associated with senescence in plants [87], increasing the activities of POD and CAT antioxidant enzymes and other resistance markers only in the late stage of storage by the CS/GA-MeSA 1 mM treatment may be valuable for increasing the postharvest shelf life of tomato fruit. Furthermore, the CS/GA-MeSA 1 mM formulation can also inhibit pathogen infection by *A. alternata* and *B. cinerea* (Figure 4A,B), demonstrating that 1 mM MeSA in the CS/GA film is a prospective applicant as a crucial signal in the formation of systemic acquired resistance [88]. Recently, extending the shelf-life period of green-stage tomato fruit by preharvest salicylic acid treatment has been previously reported [89]. They showed that 4 mM SA can induce the activity of POD compared to control, while Li et al. [90] said that 0.1 mM MeSA-pretreated fresh-cut pitaya fruit demonstrated a higher activity of PAL related to phenolic biosynthesis and the lower activities of PPO and POD closely related to phenolic oxidation compared to uncoated fruits.

The enzymatic reaction that induces browning is an ordinary incident that can be observed visibly in some postharvested fruits, which mostly affects their quality features including nutritional quality. Polyphenols were oxidized by PPO into related quinones, which might further oxidize into melanin pigment accountable for the enzymatic browning of the fruits due to excess PPO activity. PAL and PPO’s activities generally affected the load of TP and the degree of the enzymatic browning of fruits. In this study, CS/GA-SNP 2 mM caused a huge induction of browning enzymes as it had the highest PAL and PPO activities in the early stage of storage. Fortunately, this huge increase in browning enzymes in the CS/GA-SNP 2 mM treatment on the fifth day of storage was accompanied by an elevated value of TP, which will fight the proper increase in MDA, especially after the 10th day of ripening, but might induce the senescent spotting of the fruits. The lower PAL/PPO activity ratio in the CS/GA-MeSA 1 mM treatment in the early stage of ripening caused lower values of the peak maximum of browning enzymes in later stages of storage in comparison to other polymer blends incorporated by the used elicitors, consequently minimizing the impact of browning on the fruits. Overall, the pattern of SNP and MeSA elicitors in the CS/GA blend on browning enzymes disagreed with that obtained by Taher and Elsherbiny [7] for isonicotinic acid. They found that INA in the CS/PVA blends significantly reduced the activity of PAL and PPO enzymes than those of the control throughout the storage period of tomato fruits.

## 4. Conclusions

In the present study, the CS/GA blend, combined with SNP or MeSA, was excellently fabricated and applied as a coating on green tomato fruits to evaluate its beneficial impacts throughout the postharvest period. MeSA effectively increased the antifungal properties of the CS/GA blend. The outcomes obtained from this work showed that tomatoes treated by CS/GA enriched with MeSA at a concentration of 1 mM had a significant impact on hindering softening, carotenoid accumulation, and TA. The same treatment was able to fight MDA increasing and had higher contents of TPs and higher activities of POD, PPO, PAL, and CAT at the late stage of storage. Previous observations have indicated that applying a 1 mM CS/GA-MeSA formulation on freshly harvested tomato fruits in new desert lands supports their good supply to old local markets without over-maturity, skin browning, or fungal infections.

## Data Availability

All of the data are included in the article.
